# Ginsenoside-Mediated Ferroptosis Regulation: Bidirectional Effects and Therapeutic Potential in Diseases

**DOI:** 10.3390/ijms27073172

**Published:** 2026-03-31

**Authors:** Yuanyuan Wang, Mengxue Song, Shuai Li, Huizhen Ren, Shuang Liu, Hui Zhang

**Affiliations:** 1 School of Basic Medicine, Jiamusi University, Jiamusi 154007, China; wyuanyuan202303@163.com (Y.W.); 15686061872@163.com (S.L.); 18735054637@163.com (H.R.); 2 School of Clinical Medicine, Jiamusi University, Jiamusi 154007, China; 18212685516@163.com

**Keywords:** ginseng, ginsenoside, ferroptosis, cancer, non-cancer disease

## Abstract

Notably, certain ginsenoside components exhibit distinct bidirectional and context-dependent regulatory effects on ferroptosis depending on the disease setting. In aberrantly proliferating or activated cells, ginsenosides function as ferroptosis inducers, whereas in damaged quiescent cells of normal tissues, they act as ferroptosis inhibitors. The pro-ferroptotic effect is predominantly observed in cells characterized by abnormal proliferation or activation, such as cancer cells and activated hepatic stellate cells in liver fibrosis. In this context, ginsenosides modulate key iron metabolism proteins and suppress antioxidant defense systems (e.g., GPX4, SLC7A11), thereby triggering intracellular iron overload and explosive lipid peroxidation, ultimately culminating in ferroptosis. Conversely, the anti-ferroptotic effect primarily targets damaged non-proliferative cells in normal tissues subjected to pathological insults (e.g., ischemia–reperfusion, inflammation). In this setting, the regulatory focus of ginsenosides shifts toward maintaining iron homeostasis through mechanisms including upregulation of iron storage proteins (e.g., FTH1), downregulation of iron uptake proteins (e.g., TFRC), and inhibition of labile Fe^2+^ accumulation, thereby blocking ferroptosis initiation. This review systematically elucidates the pharmacological effects and underlying mechanisms by which different ginsenoside components regulate ferroptosis across various disease contexts and cell types, with particular emphasis on their disease- and cell type-dependent bidirectional regulatory characteristics. By highlighting these context-specific effects, we aim to provide novel potential therapeutic targets and mechanistic insights for the precision treatment of diverse pathological conditions, including malignant proliferative disorders, non-malignant aberrantly activated/proliferative diseases such as liver fibrosis, and cell injury/degenerative diseases.

## 1. Introduction

**Figure 1:**
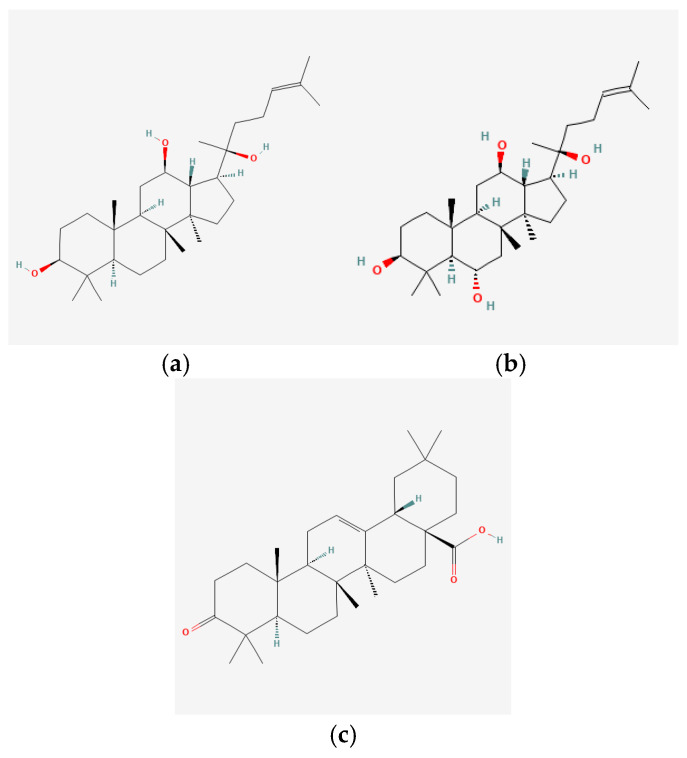
The structure of ginsenosides: (**a**) Protopanaxadiol-type ginsenosides (20(S)-protopanaxadiol). (**b**) Protopanaxatriol-type ginsenosides (20(S)-protopanaxatriol). (**c**) Oleanane-type ginsenosides.

In recent years, Traditional Chinese medicine (TCM) has attracted extensive research attention due to its broad spectrum of pharmacological activities, remarkable therapeutic efficacy, and favorable safety profile. Beyond multi-herb compound prescriptions, TCM also includes diverse individual active components. These exhibit remarkable chemical structural diversity and demonstrate extensive biological activities [1]. Previous studies have shown that several traditional Chinese medicines can effectively inhibit the proliferation of tumor cells by regulating cell death mechanisms such as pyroptosis and apoptosis [2]. Notably, accumulating evidence suggests that some TCMs can further influence the development of diverse diseases through the modulation of ferroptosis [3], and ginseng stands out as a prominent representative among such natural medicinal materials. Regarded as a precious medicinal herb in TCM, ginseng exhibits significant therapeutic efficacy. Ginsenosides, its major active components, are triterpenoid saponins [4,5]. So far, more than 500 distinct ginsenosides have been isolated and characterized from ginseng [6]. Based on their aglycone skeletons, these compounds are classified into three main categories: protopanaxadiol-type (e.g., Rb1, Rb2, Rd, Rg3, Rg5; RK1; Rh2; CK), protopanaxatriol-type (e.g., Re, Rg1, Rg2, Rf, Rh1; Rh3; Rh4), and oleanane-type (Ro) (Figure 1a–c). Derived from the long-standing application of ginseng in TCM, ginsenosides have been used clinically for treating diverse diseases for over 100 years [7]. Accumulating evidence indicates that ginsenosides exhibit multiple therapeutic properties for treating diverse diseases, including anti-inflammatory, anti-tumor, and immunomodulatory effects [8,9,10]. Furthermore, individual ginsenosides are not specific to a single tumor type and instead display extensive anti-tumor activity. For example, ginsenoside Rg3 (GRg3) reverses cisplatin resistance in non-small cell lung cancer (NSCLC) by suppressing chemotherapy-induced Programmed Death-Ligand 1 (PD-L1) upregulation [11], while attenuating the progression of malignant osteosarcoma via the circular RNA 0003074 (circ-0003074)/microRNA-516b-5p (miR-516b-5p)/Karyopherin Alpha 4 (KPNA4) axis [12]. Collectively, these findings highlight that ginsenosides have emerged as natural products with substantial clinical potential, holding promising therapeutic prospects for treating various complex diseases. Moreover, a growing body of research suggests that ginsenosides interact closely with ferroptosis, which can influence cellular fate and disease progression by regulating ferroptosis, potentially through positive feedback loops. Defined as an iron-dependent, non-apoptotic form of cell death, ferroptosis was first proposed in 2012 after being identified in oncogenic RAS-mutated tumor cells, and is implicated in the pathogenesis of multiple diseases [13,14]. Distinguished from programmed cell death (PCD) types, including autophagy, apoptosis, and pyroptosis, ferroptosis is defined by typical features: glutathione (GSH) exhaustion, malondialdehyde (MDA) production, iron overload, increased lipid peroxidation, and excessive reactive oxygen species (ROS) accumulation [15,16,17,18]. Therefore, the detection of MDA, Fe^2+^, ROS, GSH and glutathione peroxidase 4 (GPX4) expression is commonly employed to assess ferroptosis. Notably, ferroptosis exerts a critical function in tumorigenesis, tumor immunity, and the efficacy of chemotherapy and radiotherapy [19,20,21]. It is detectable in various tumor cell types and regulated by numerous factors, including transcriptional regulation and cellular energy metabolism. Thus, ferroptosis is recognized as an endogenous tumor suppressor mechanism [22]. Inducing ferroptosis can effectively suppress tumor cell proliferation, metastasis and chemoresistance. Therefore, targeted modulation of the ferroptosis pathway is gradually gaining recognition as a highly promising cancer treatment strategy [23,24,25,26]. Existing research indicates that ferroptosis not only holds potential as a breakthrough in anti-tumor therapy but also offers a means to address the limitations of conventional chemotherapy and classic targeted drugs [27], thereby opening up novel avenues for its clinical translation in tumor treatment. Thus, developing drugs that target ferroptosis mechanisms has become an important focus in the field of anti-cancer therapy. Furthermore, ferroptosis research is not confined to cancer; in recent years, relevant studies have been increasingly conducted on ferroptosis in various non-cancerous conditions, including liver fibrosis, neurodegenerative diseases, and ischemia–reperfusion injury [28,29,30]. TCM exhibits unique advantages in the bidirectional modulation of ferroptosis via multi-gene targeted regulation [31,32], and this regulatory mechanism offers substantial potential to establish novel therapeutic avenues for the clinical management of diverse human diseases. The primary aim of this review is to elaborate on the bidirectional regulatory effects of ginsenosides on ferroptosis: they induce ferroptosis to inhibit tumor cell viability in tumor contexts, while conversely suppressing ferroptosis in disease models, including neurodegenerative disorders, to afford protective effects for normal cells. Of importance, in liver fibrosis models, ginsenosides exert analogous activity to their action in tumor models, inducing ferroptosis to suppress the activation of hepatic stellate cells (HSCs) with attendant attenuation of fibrotic progression. This hallmark of bidirectional regulation not only highlights the dual functional role of ferroptosis across distinct disease settings but also confirms that individual ginsenoside constituents exert divergent biological effects under specific disease microenvironments and cellular subtypes. This regulatory paradigm achieves disease progression alleviation in turn and establishes a robust theoretical basis to support the clinical translational development of novel targeted therapeutic agents.

Of particular note, existing research has confirmed that ginsenosides mediate bidirectional regulation of ferroptosis, yet several critical questions remain to be elucidated. For instance, why do identical ginsenoside components (e.g., GRg3, Ginsenoside Rh2(GRh2)) exert opposing ferroptosis-regulatory effects across distinct disease models or cellular subtypes? Might structural differences in the specific chemical moieties of ginsenoside constituents be closely correlated with their capacity to modulate ferroptosis? In parallel, the majority of current investigations remain confined to cellular and animal experimental models, and the clinical translational potential of ginsenosides as ferroptosis modulators requires further in-depth exploration. Based on current research evidence, this review elaborates on the bidirectional regulatory effects of ginsenosides on ferroptosis in the context of diverse disease states.

## 2. The Regulation of Ferroptosis by Ginsenosides in Diseases

### 2.1. Diseases Characterized by Malignant Cellular Proliferation

**Figure 2:**
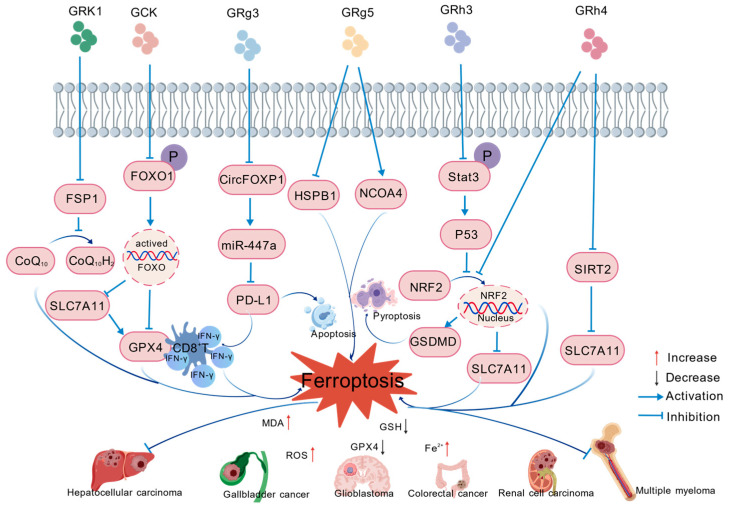
Mechanism by which specific ginsenoside components induce ferroptosis in cancer cells by regulating multiple signaling pathways. GRK1: ginsenoside RK1. GCK: ginsenoside CK. GRg3: ginsenoside Rg3. GRg5: ginsenoside Rg5. GRh3: ginsenoside Rh3. GRh4: ginsenoside Rh4. FSP1: ferroptosis suppressor protein 1. CoQ_10_: Coenzyme Q_10_. CoQ_10_H_2_: Coenzyme Q_10_ hydroquinone. FOXO1: Forkhead Box O1. SLC7A11: solute family 7 member 11. GPX4: glutathione peroxidase 4. miR-447a: microRNA-447a. PD-L1: Programmed Death-Ligand 1. HSPB1: heat shock protein family B (small) member 1. NCOA4: nuclear receptor coactivator 4. Stat3: Signal Transducer and Activator of Transcription 3. p53: Tumor Protein p53. NRF2: nuclear factor erythroid 2-related factor 2. GSDMD: gasdermin D. SIRT2: sirtuin 2. MDA: malondialdehyde. ROS: reactive oxygen species. GSH: glutathione.

Across diverse cancer models, specific ginsenoside constituents suppress tumor cell proliferation and migratory activity via ferroptosis induction. These compounds selectively modulate distinct signaling pathways, eliciting dysfunction of the intracellular antioxidant defense system and excessive lipid peroxidation accumulation, with consequent induction of ferroptotic cell death (Table 1, Figure 2). These studies elucidate the key mechanistic underpinnings by which ginsenosides act as ferroptosis inducers for cancer therapy, highlighting their extensive clinical application potential.

#### 2.1.1. Glioblastoma

Glioblastoma, a highly malignant neoplasm, is distinguished by rapid growth, strong invasiveness, pronounced chemoresistance, and substantial heterogeneity [40], which presents a major challenge to clinical treatment. One critical reason for the difficulty in glioma therapy is the poor blood–brain barrier penetration of most anti-tumor drugs, leading to inadequate efficacy of systemic treatments and driving the search for novel therapeutic approaches. Formed by the steaming of ginseng, ginsenoside Rg5 (GRg5) is produced through the deglycosylation of ginsenoside Rb1 (GRb1) and dehydration at the C-20 position of ginsenoside Rg3 (GRg3). Relative to its main derivatives, GRg5 demonstrates superior pharmacological effects [41]. As a protopanaxadiol-type ginsenoside, the dehydration modification at the C-20 position of GRg5 enhances its lipid solubility and binding affinity to nuclear receptors, which is a key structural basis for its specific regulation of the NR3C1/HSPB1/NCOA4 axis. It is worth emphasizing that GRg5 exhibits inhibitory effects on the progression of diverse malignancies, including retinoblastoma, breast cancer and gastric cancer [42,43,44]. Specifically, GRg5 mediates bidirectional regulation of core ferroptosis-related molecules through direct binding to and consequent upregulation of nuclear receptor subfamily 3, group C, member 1 (NR3C1)—a well-established transcription factor. NR3C1 exerts contrasting transcriptional control over two ferroptosis-related molecules: it downregulates transcription of heat shock protein family B (small) member 1 (HSPB1)—a known ferroptosis suppressor—yet boosts transcription of nuclear coactivator 4 (NCOA4) [38]. NCOA4—a key mediator of ferritinophagy—promotes ferritin degradation in autophagolysosomes, triggering robust release of intracellular free iron. This sequential process triggers marked lipid peroxidation, culminating in ferroptosis of glioma stem cells.

This mechanism exemplifies how a ginsenoside can directly modulate an upstream transcription factor (NR3C1) to simultaneously control both the iron storage pathway (via NCOA4) and the cytoskeletal response to stress (via HSPB1), ultimately converging on the hallmark features of ferroptosis: iron dyshomeostasis and lipid peroxidation.

Experimental validation—both in vitro and in vivo—confirms this mechanism, while also supporting GRg5’s inhibitory effects on malignant phenotypes and disease progression. These findings provide critical theoretical support for advancing GRg5’s clinical translation as a potential glioblastoma therapeutic, centered on ferroptosis induction.

#### 2.1.2. Hepatocellular Carcinoma (HCC)

HCC is a prevalent malignant tumor of the digestive system with high incidence and mortality rates [45], posing a severe threat to human health. Characterized by insidious onset, rapid progression, late diagnosis, and high recurrence rate, HCC remains a major clinical challenge. Although surgical resection and liver transplantation have proven therapeutic efficacy, identifying effective therapeutic targets and agents remains critical to addressing the hurdles in HCC treatment [46]. In recent years, ferroptosis has been recognized as a potential therapeutic target for HCC [47]. Ginsenoside RK1 (GRK1) is a rare ginsenoside isolated from ginseng. It effectively induces G0/G1 phase cell cycle arrest, impairs the migration, proliferation, and invasion of cancer cells, and subsequently reduces the risk of metastasis [48]. Multiple studies have confirmed that GRK1 exerts significant anti-tumor effects against various malignancies, including liver cancer and lung cancer [49,50], and its mechanism of action involves multiple regulatory pathways [51,52]. GRK1 significantly downregulates the expression of ferroptosis suppressor protein 1 (FSP1) [34], thereby disrupting the ferroptosis defense system in HCC cells. Specifically, GRK1 reduces intracellular GSH levels, concurrently elevates intracellular lipid peroxidation levels and elicits a pronounced elevation in intracellular iron ion concentrations; these concerted actions trigger ferroptosis in HCC cells and exert marked attenuation on their proliferative capacity and survival viability. In addition to GRK1, ginsenoside compound K (GCK)—a further bioactive constituent—also possesses diverse pharmacologic activities against HCC, encompassing anti-inflammatory, anti-tumor, hepatoprotective and immunomodulatory properties [53,54,55,56]. GCK shares a strong regulatory association with the phosphatidylinositol 3-kinase (PI3K)/protein kinase B (AKT) signaling pathway; a growing body of research has verified that GCK mediates anti-tumor effects via targeted inhibition of the PI3K/AKT pathway [57], thereby curtailing tumor cell proliferation and eliciting apoptotic cascades in malignant HCC cells. This pathway inhibition further restricts tumor cell growth progression and induces programmed apoptotic death in tumor cells. Concurrently, GCK attenuates phosphorylation of Forkhead Box O1 (FOXO1) and initiates activation of the FOXO signaling pathway [35], in turn reducing the expression levels of solute carrier family 7 member 11 (SLC7A11) and GPX4—two core regulatory proteins governing ferroptosis.

It is noteworthy that while GRK1 targets the FSP1-CoQ10 system and GCK targets the PI3K/AKT/FOXO axis, both interventions lead to a common terminal outcome: the depletion of GSH and the accumulation of lipid peroxides. This suggests that the PI3K/AKT pathway may serve as a context-dependent transducer of a more fundamental metabolic stress signal initiated by GCK. Such impairment of the cellular antioxidant defense system elicits intracellular GSH depletion and progressive accumulation of lipid peroxidation, and culminates in ferroptosis induction in HCC cells alongside suppressed malignant proliferation, which establishes a promising targeted therapeutic strategy for HCC intervention.

#### 2.1.3. Gallbladder Cancer (GBC)

GBC is the most common malignant neoplasm of the biliary tract and constitutes a primary contributor to cancer-associated mortality worldwide [58]. This malignancy possesses strong invasive potential, and as yet, no specific therapeutic agents have been clinically approved for its management—a factor that translates to dismal overall clinical prognosis in affected patients. Within anti-tumor research contexts, ginsenoside Rg3 (GRg3) has been validated to potently trigger tumor cell apoptosis, suppress malignant cell proliferation and invasive activity, and impede tumor angiogenesis; this multifaceted anti-tumor action in turn mitigates the likelihood of distant metastatic spread and tumor recurrence [59]. In addition to this, GRg3 harbors distinct immunomodulatory activity [60]. Augmentation of immune effector functions in CD8-positive T (CD8^+^T) cells represents an important therapeutic strategy for improving clinical management of GBC. Wu et al. [61] demonstrated that GRg3 modulates the CREBRF-CREB3 signaling pathway to suppress autophagic activity, in turn augmenting the sensitivity of tumor cells to chemotherapeutic agents; GRg3 also enhances activation of the endoplasmic reticulum stress (ERS)-mediated signaling pathway to promote apoptosis in cancer cells [62]. It is worth highlighting that Ye et al. [36] identified that GRg3 restores the biological activity of CD8^+^T cells via suppression of the circular RNA FOXP1 (circFOXP1)/microRNA-447a (miR-447a)/PD-L1 signaling axis. Moreover, this cellular regulatory cascade coincides with the induction of ferroptosis and apoptosis in GBC cells; this dual cell death process suppresses cancer cell proliferative potential and yields beneficial therapeutic responses for GBC treatment.

The involvement of the circFOXP1/miR-447a/PD-L1 axis in GRg3-mediated ferroptosis is particularly intriguing, as it links the core cell death mechanism to the immune microenvironment. It raises the possibility that the changes in iron and ROS levels induced by GRg3 may act as an upstream signal that modulates both cancer cell intrinsic survival and extrinsic immune recognition. Collectively, these investigations verify GRg3 triggers ferroptosis to inhibit cancer cell proliferation and migration, ultimately conferring effective therapeutic activity against GBC.

#### 2.1.4. Renal Cell Carcinoma (RCC)

RCC is a common malignant neoplasm of the urinary system [63], carrying high incidence and mortality rates that pose a serious threat to patient health. Mounting research confirms that inducing ferroptosis stands as a promising new therapeutic approach for treating diverse cancers, including RCC [64]. Ginsenoside Rh4 (GRh4) is a relatively uncommon bioactive constituent isolated from ginseng. Studies have confirmed that GRh4 exerts tumor-suppressive effects in multiple malignancies, including esophageal cancer [65] and gastric cancer [66]. Li et al. [67] reported that aberrant activation of the nuclear factor erythroid 2-related factor 2 (NRF2) signaling pathway is closely associated with the progression of various malignant tumors, including RCC. Zhao et al. [37] demonstrated that GRh4 significantly downregulates the expression of NRF2-regulated key antioxidant proteins (such as GPX4) by inhibiting the NRF2 signaling pathway. This impairs tumor cells’ antioxidant defense ability, in turn greatly boosting RCC cells’ sensitivity to ferroptosis.

The NRF2 pathway acts as a master regulator of the antioxidant response. By inhibiting NRF2, GRh4 effectively lowers the threshold for lipid peroxidation, demonstrating how targeting a single upstream hub can sensitize cells to ferroptosis triggered by endogenous metabolic byproducts. This mechanism underscores GRh4’s potential as an adjuvant therapeutic for RCC—especially in combination with ferroptosis inducers and offers critical theoretical backing for the clinical use of GRh4 paired with ferroptosis inducers in RCC therapy.

#### 2.1.5. Colorectal Cancer (CRC)

CRC is a prevalent malignant tumor of the digestive system [68]. Characterized by insidious and atypical early symptoms, most patients are diagnosed at an advanced stage when CRC is identified. Currently, the main clinical treatments remain surgery, neoadjuvant radiotherapy, and adjuvant chemotherapy [69]. However, chemoresistance frequently develops in some patients during treatment, compromising therapeutic efficacy. The anti-apoptotic mechanism of tumor cells is one of the key factors contributing to chemoresistance. Therefore, screening natural products for drugs targeting non-apoptotic cell death pathways is of great significance, as it may provide a novel direction for overcoming chemoresistance. Wu et al. [38] demonstrated that ginsenoside Rh3 (GRh3) not only activates the gasdermin D (GSDMD)-dependent pyroptosis pathway but also inhibits the nuclear translocation of NRF2 by regulating the Signal Transducer and Activator of Transcription 3 (Stat3)/Tumor Protein p53 (p53)/NRF2 signaling axis. The latter effect leads to reduced activity of SLC7A11 and GPX4, accompanied by GSH depletion and accumulation of iron, lipid ROS, and MDA, ultimately inducing ferroptosis in CRC cells. Notably, this mechanism exerts minimal effects on normal cells, highlighting Rh3’s promising anti-cancer potential.

The concurrent induction of pyroptosis and ferroptosis by GRh3 via the Stat3/p53/NRF2 axis illustrates a crucial point: ginsenosides often target pleiotropic upstream regulators that control multiple downstream cell death modalities. This crosstalk suggests that the “ferroptosis” observed may be part of a broader cellular disintegration program triggered by the collapse of antioxidant defenses. Collectively, GRh3 can effectively inhibit CRC cell proliferation by synergistically activating the dual cell death pathways of pyroptosis and ferroptosis, providing a potential direction for drug development to address chemoresistance.

#### 2.1.6. Multiple Myeloma (MM)

MM is a malignant hematological tumor characterized by plasma cell terminal differentiation [70]. Owing to its unclear pathogenesis and limited therapeutic options, the current clinical efficacy remains unsatisfactory, with patients maintaining a relatively high mortality rate [71]. GRh4, a triol-type ginsenoside with low polarity, exhibits favorable intestinal absorption properties and high bioavailability [72]. It exerts significant therapeutic effects against various malignancies. In MM, GRh4 can directly bind to and inhibit sirtuin 2 (SIRT2) [39], which in turn downregulates the expression of key iron metabolism and antioxidant proteins, including SLC7A11, GPX4, and ferritin heavy chain 1 (FTH1). This impairs the antioxidant defense system of MM cells. Meanwhile, GRh4 upregulates the expression of long-chain acyl-CoA synthase 4 (ACSL4), increases lipid peroxidation substrates, and promotes the accumulation of lipid ROS, ultimately inducing ferroptosis in MM cells and suppressing the malignant progression of MM. By simultaneously inhibiting antioxidant systems (SLC7A11/GPX4) and promoting the biosynthesis of peroxidation-sensitive lipids (via ACSL4), GRh4 acts on two parallel and synergistic arms of ferroptosis regulation, showcasing a multi-pronged attack on a shared vulnerability—the lipid peroxide repair capacity of the cell.

### 2.2. Non-Malignant Diseases Characterized by Abnormal Cellular Activation and Proliferation

**Figure 3:**
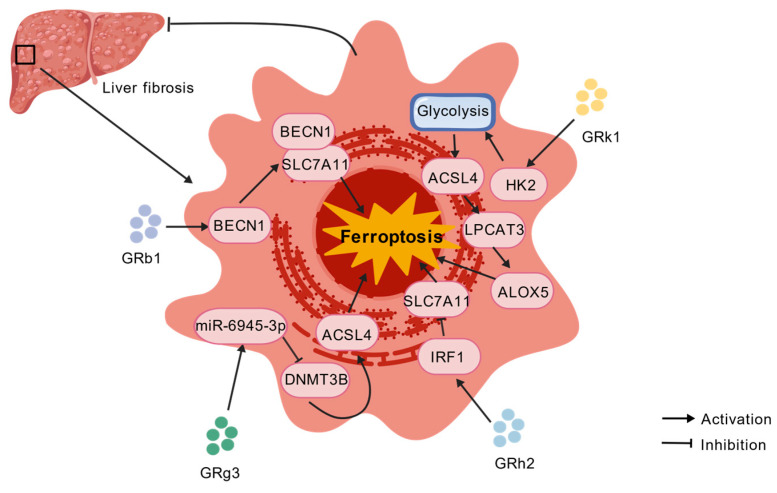
Mechanism by which ginsenosides induce ferroptosis of hepatic stellate cells through multi-pathway synergistic action to improve liver fibrosis. GRb1: ginsenoside Rb1. GRg3: ginsenoside Rg3. GRh2: ginsenoside Rh2. GRk1: ginsenoside Rk1. BECN1: Beclin-1. SLC7A11: solute family 7 member 11. miR-6945-3p: microRNA-6945-3p. DNMT3B: DNA methyltransferase 3B. ACSL4: long-chain acyl-CoA synthase 4. IRF1: interferon regulatory factor 1. HK2: hexokinase 2. LPCAT3: Lysophosphatidylcholine Acyltransferase 3. ALOX5: Arachidonate Lipoxygenase 5.

**Figure 4:**
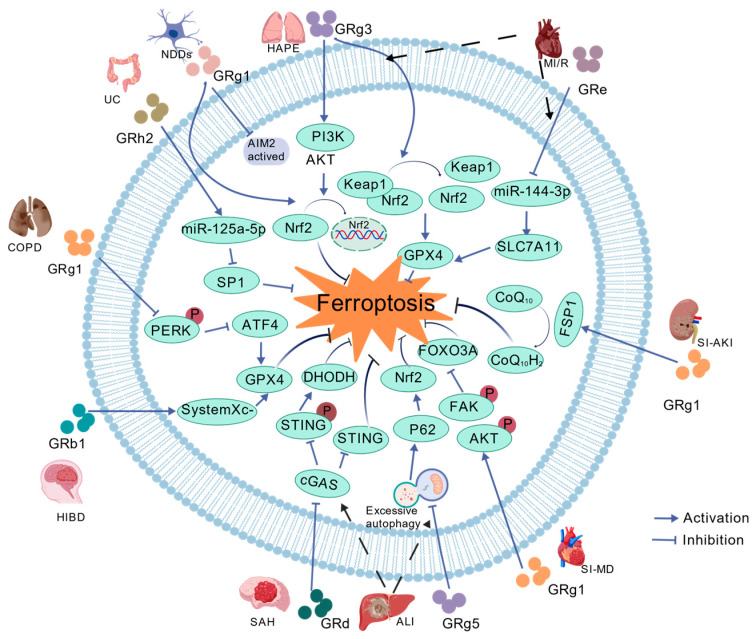
Mechanism by which specific ginsenoside components inhibit ferroptosis in damaged tissues and organs by regulating multiple signaling pathways. HAPE: High-altitude pulmonary edema. NDDs: neurodegenerative diseases. UC: ulcerative colitis. COPD: chronic inflammatory neurodegenerative disease. HIBD: hypoxic–ischemic brain injury. SAH: subarachnoid hemorrhage. ALI: acute liver injury. SI-MD: sepsis-induced myocardial dysfunction. SI-AKI: sepsis-induced kidney injury. MI/R: myocardial ischemia/reperfusion. GRg3: ginsenoside Rg3. GRg1: ginsenoside Rg1. GRh2: ginsenoside Rh2. GRb1: ginsenoside Rb1. GRd: ginsenoside Rd. GRg5: ginsenoside Rg5. GRe: ginsenoside Re. PI3K: phosphatidylinositol 3-kinase. Keap1: Kelch-like ECH-associated protein 1. NRF2: nuclear factor erythroid 2-related factor 2. GPX4: glutathione peroxidase 4. miR-144-3p: mi-croRNA-144-3p. SLC7A11: solute family 7 member 11. FSPI: ferroptosis suppressor protein 1. CoQ_10_: coenzymeQ_10_. CoQ_10_H_2_: Coenzyme Q_10_ dihydroquinone. AKT: protein kinase B. FAK: focal adhesion kinase. FOXO3A: Forkhead Box O3A. p62/SQSTM1: sequestosome 1. cGAS: cyclic GMP-AMP Synthase. STING: Stimulator of Interferon Genes. DHODH: dihydroorotate dehydrogenase. PERK: protein kinase R-like endoplasmic reticulum kinase. ATF4: activating transcription factor 4. SP1: specificity protein 1. miR-125a-5p: microRNA-125a-5p.

The therapeutic potential of ginsenosides covers a wide range of diseases, exerting favorable therapeutic effects in all these conditions. Malignant cell proliferation-related diseases are represented by various types of cancer, while non-malignant abnormal activation and proliferation-related diseases are typically exemplified by hepatic fibrosis. In such diseases, the mechanism of action of ginsenosides is similar to that observed in cancer settings. Hepatic stellate cells (HSCs) serve as the core effector cells in the progression of hepatic fibrosis, and their abnormal activation acts as a key driver of disease advancement; ginsenosides can effectively inhibit the progression of hepatic fibrosis by regulating iron metabolic homeostasis, activating lipid peroxidation pathways, and inducing ferroptosis in these cells (Table 2 and Figure 3). In contrast, in cell injury/degeneration-related diseases, ischemia–reperfusion injury and sepsis-associated organ injury are the most typical examples. Ginsenosides adopt a different mechanism of action from that in the previous two types of diseases. They regulate relevant signaling pathways to restrict intracellular iron overload and reduce reactive oxygen species (ROS) accumulation, thereby blocking the ferroptosis process and exerting protective effects on damaged tissues and organs (Table 3 and Figure 4).

Collectively, these research findings demonstrate that ginsenosides exert a significant disease context-dependent bidirectional regulatory effect on ferroptosis. This refined regulatory pattern provides a core mechanistic basis for their targeted application in various diseases, and also lays a theoretical foundation for the targeted protection and functional restoration of damaged tissues.

#### Liver Fibrosis

In the uninjured liver, HSCs occupy the Space of Disse as quiescent cells specialized for vitamin A storage; conversely, persistent parenchymal damage drives their transdifferentiation into myofibroblasts, marked by de novo α-smooth muscle actin (α-SMA) expression, migration to injured foci, and robust secretion of extracellular matrix (ECM) [88,89]. Sustained activation of HSCs constitutes the primary pathogenic driver of liver fibrosis and the pathological process is closely correlated with ferroptosis. As a key driver of liver disease progression, fibrosis profoundly impacts prognosis and HCC risk [90]. Timely intervention and removal of the causative factors may reverse early liver fibrosis. Nevertheless, such reversal is frequently slow and inadequate, especially in patients with advanced fibrosis. Thus, there is a considerable unmet medical need for anti-fibrotic therapies to prevent liver disease progression and HCC development. Accumulating evidence confirms that ginsenosides differing in chemical structure trigger ferroptosis in HSCs through a variety of mechanisms, in turn dampening HSC activation. Protopanaxadiol-type ginsenosides GRg3 [91,92,93], GRh2 [94], and GRb1 [95] have been verified to exert significant antitumor, anti-inflammatory, and hepatoprotective effects. The rare ginsenoside RK1, obtained from ginseng via deglycosylation, also exhibits diverse biological activities. It not only possesses antitumor properties [52,96] but also shows distinctive application potential in the treatment of hepatic fibrosis [51]. All four aforementioned ginsenosides can suppress the activation of hepatic stellate cells (HSCs) and delay the progression of hepatic fibrosis, yet they differ markedly in the signaling pathways and target genes responsible for their biological effects. ACSL4 participates in the conversion of fatty acids to fatty acyl-CoA esters and triggers ferroptosis through the regulation of lipid biosynthesis [97] acting in turn as a core regulatory molecule in ferroptosis execution. Studies have confirmed that GRg3 inhibits DNA methyltransferase 3B (DNMT3B) activity through miR-6945-3p-mediated epigenetic regulation [98]. This suppressive action depends on blocking methylation within the ACSL4 gene promoter region, reinstating ACSL4 protein expression as a result. Elevated ACSL4 expression notably enhances ferroptosis sensitivity in HSCs, in turn promoting HSC inactivation.

Findings from Lin and colleagues [73] demonstrated that GRb1 boosts the expression of the autophagy-associated protein Beclin 1 (BECN1) and reinforces the interaction between BECN1 and SLC7A11, facilitating their assembly into the BECN1-SLC7A11 complex. The GRb1-driven BECN1/SLC7A11 axis engages with the autophagy pathway: GRb1 initiates ferroptosis by downregulating SLC7A11 expression, while increasing autophagy flux, consequently forming a ferroptosis-autophagy synergism that diminishes HSC activation. This then brings about reduced SLC7A11 protein expression, reducing SLC7A11-mediated cystine uptake, disrupting the intracellular antioxidant defense system, and inducing ferroptosis in HSCs—ultimately imparting a notable mitigating effect on liver fibrosis progression.

Lang et al. [74] demonstrated that GRh2 boosts the expression of interferon regulatory factor 1 (IRF1) and downregulates its downstream target gene SLC7A11—triggering ferroptosis and ensuing HSC inactivation, while ultimately mitigating hepatic inflammatory responses and improving liver fibrosis. Apart from the ferroptosis pathway, GRh2 also suppresses liver fibrosis progression through other mechanisms. Chen et al. [99] showed that GRh2 effectively governs HSC activation and proliferation by blocking autophagy, thereby slowing liver fibrosis progression.

One core regulatory pathway governing ferroptosis corresponds to the ACSL4/lysophosphatidylcholine acyltransferase 3 (LPCAT3)/arachidonate lipoxygenase (ALOX) signaling axis [100]; its activation prompts lipid peroxidation, elevates intracellular ROS production, and ultimately initiates cellular ferroptosis. Xia et al. [75] confirm that GRK1 improves liver fibrosis through activation of the ACSL4/LPCAT3/ALOX5 signaling axis. Of note, GRK1 binds directly to hexokinase 2 (HK2)—a key rate-limiting enzyme in glycolysis—stabilizes its protein structure and delays its degradation, in turn fostering intracellular HK2 accumulation. Augmented HK2 goes on to boost ACSL4 expression, while simultaneously enhancing its mRNA abundance and protein production. Following this, increased ACSL4 intensifies lipid peroxidation through the LPCAT3/ALOX5 pathway and triggers ferroptosis in HSCs, in turn significantly dampening HSCs activation.

Across these studies, a consistent theme emerges: ginsenosides induce HSC ferroptosis by converging on the ACSL4-driven lipid peroxidation pathway or the SLC7A11/GPX4 antioxidant axis, regardless of whether the initiating signal is epigenetic (GRg3), autophagic (GRb1), transcriptional (GRh2), or metabolic (GRk1). This reinforces the concept that these pathways are not isolated but rather represent different routes to the same ferroptosis endpoint.

### 2.3. Diseases Characterized by Cellular Injury and Degeneration

#### 2.3.1. Acute Liver Injury (ALI)

ALI is a severe hepatic parenchymal lesion, and its etiologies involve various categories, including drug-induced hepatotoxicity, chemical exposure, chronic excessive alcohol intake and infectious pathogens. Accumulating evidence verifies that NRF2 plays a critical regulatory role in ferroptosis; it regulates genes linked to heme synthesis, hemoglobin catabolism, and iron storage and export [101], and acts as a principal regulatory molecule for maintaining iron metabolic homeostasis within the organism. NRF2 binds to antioxidant response elements (AREs) and triggers transcriptional expression of downstream antioxidant target genes, with resultant alleviation of oxidative stress-induced cellular damage [102]. It is worth noting that activation of the NRF2-GPX4 signaling axis effectively attenuates excessive ROS accumulation and further suppresses ferroptosis [103]. In a lipopolysaccharide (LPS)-induced ALI model, researchers have identified marked ROS overproduction in vivo, which provokes a robust oxidative stress response and is coupled with aberrantly heightened autophagy activity. Excessive autophagic activation directly suppresses NRF2 activity [104], with resultant substantial reductions in the expression levels of its downstream antioxidant proteins (heme oxygenase-1 (HO-1) and GPX4) and iron metabolism-associated proteins (FTH1 and FSP1). These alterations act in a synergistic manner and go on to induce intracellular iron accumulation plus lipid peroxidation, with subsequent acceleration of ferroptosis and exacerbation of hepatocellular injury. Pertinent research findings verify that GRg5 regulates hyperactivated autophagy through upregulation of Sequestosome 1 (SQSTM1/p62) and Microtubule-associated Protein 1 Light Chain 3A/B (LC3A/B) expression [76], with concomitant activation of the NRF2 signaling pathway. This elevates the expression levels of HO-1, GPX4 and iron metabolism-related proteins, curtails intracellular iron accumulation and excessive ROS generation, and suppresses ferroptosis, with resultant substantial remission of LPS-induced ALI. In comparison with other ginsenosides, ginsenoside Rd (GRd) contains an additional xylose molecule within its chemical structure (a protopanaxadiol-type ginsenoside with three sugar chains), and its marked anti-inflammatory, antioxidant and anti-apoptotic activities have been validated in experimental studies [105,106,107]. In a carbon tetrachloride (CCl_4_)-induced ALI mouse model, GRd attenuates ferroptosis by suppressing activation of the cyclic GMP-AMP synthase (cGAS)/stimulator of interferon genes (STING) signaling pathway [77], and in parallel upregulates the expression of GSH and GPX4, with a pronounced hepatoprotective effect. Both GRg5 and GRd, despite acting on different upstream signals (autophagy vs. cGAS/STING), ultimately restore the function of the GPX4 antioxidant system, demonstrating a common mechanism of ferroptosis inhibition in the context of acute inflammation.

#### 2.3.2. Hypoxic–Ischemic Brain Damage (HIBD)

HIBD represents a primary cause of neonatal mortality worldwide and is also the most common etiology of neonatal brain injury at present [108]. This disorder imposes a serious threat to infant survival and elicits prolonged adverse impacts on neurodevelopment, with no effective therapeutic interventions available for its clinical management to date. In the pathological progression of HIBD, neonatal tissues display heightened oxidative stress, exacerbated inflammatory responses and excessive ROS generation, with subsequent induction of ferroptosis. GRb1 was highlighted in the preceding sections, and prior research has validated its protective effects on brain tissue [109] From related mechanistic research, Zhang et al. [78] demonstrated that GRb1 may curtail lipid peroxidation and inflammatory responses through activation of the System Xc^−^-GSH-GPX4 antioxidant axis. This biological action mitigates oxidative stress-mediated neuronal damage, with resultant ferroptosis suppression and concomitant protective effects on brain tissue.

#### 2.3.3. Subarachnoid Hemorrhage (SAH)

SAH has been identified as the third most common type of stroke [110], with an incidence second only to ischemic stroke and intracerebral hemorrhage. Notably, SAH can induce ferroptosis. As previously mentioned, accumulating evidence has demonstrated that GRd exerts a neuroprotective effect by preventing neuronal damage induced by cerebral ischemia–reperfusion injury [111]. In SAH models, the expression of intracellular antioxidant factors—including SLC7A11 and GPX4_is downregulated, while the expression of ACSL4 and transferrin receptor (TfR) is upregulated. These changes lead to excessive accumulation of intracellular lipid peroxides, ultimately triggering ferroptosis. Jiang et al. [79] verified that GRd inhibits the cGAS/STING/dihydroorotate dehydrogenase (DHODH) pathway, enhances mitochondrial and cellular antioxidant capacity, and reduces oxidative stress and iron accumulation. Consequently, GRd alleviates neuronal ferroptosis both in vitro and in vivo following SAH, ultimately mitigating early brain injury (EBI) post-SAH.

#### 2.3.4. Myocardial Ischemia–Reperfusion (MI/R)

Myocardial ischemia constitutes the primary pathological basis underlying the onset and progression of heart failure. Both myocardial infarction onset and the concomitant inflammatory response induce irreversible cardiac tissue injury. In clinical settings, prompt and effective myocardial reperfusion strategies, including percutaneous coronary intervention (PCI) and thrombolytic therapy, represent the principal clinical interventions for mitigating myocardial ischemic injury [112]. In fact, sustained myocardial ischemia, irrespective of reperfusion therapy implementation, can aggravate irreversible myocardial injury and induce additional cardiomyocyte apoptosis. Traditional drug therapies are unable to counteract potential injury to cardiomyocytes and the vascular system, with notable therapeutic limitations evident in their clinical application. Prior research has verified that GRg3 effectively reduces myocardial infarct size, preserves normal physiological function of the left ventricle and exerts tangible ameliorative actions against myocardial ischemia–reperfusion injury [113]. Recent experimental investigations have confirmed that GRg3 exerts robust regulatory effects in a rat model of myocardial hypertrophy established by transverse aortic coarctation (TAC), and mitigates heart failure induced by coronary artery ligation in mice [92]. These experimental observations further validate the critical pharmacological value of GRg3 in the therapy of cardiovascular diseases. Importantly, ferroptosis has been well verified to serve as a crucial contributor to the pathogenesis of myocardial ischemia/reperfusion injury [114]. Zhong et al. [80] experimentally demonstrated that GRg3 attenuates myocardial ischemia/reperfusion (MI/R)-induced ferroptosis via modulation of the Kelch-like ECH-associated protein 1 (Keap1)/NRF2/GPX4 signaling pathway, with concomitant induction of distinct cardioprotective effects. Further, a separate study has demonstrated that GRg3 attenuates mitochondrial oxidative stress injury via the Nrf2/HO-1 signaling pathway, with subsequent attenuation of cerebral ischemia/reperfusion (I/R) injury [115]. Ginsenoside Re (GRe), a major bioactive constituent of ginseng, confers protective activity against MI/R injury. Ye et al. [81] constructed a rat model of MI/R injury and identified that GRe downregulates miR-144-3p, with resultant relief of its inhibitory action on SLC7A11. This functional restoration of SLC7A11 restored cellular cystine uptake and GSH synthesis capacity, augmented GPX4 activity and suppressed lipid peroxidation events, with attendant attenuation of ferroptosis triggered by myocardial I/R injury.

#### 2.3.5. High-Altitude Pulmonary Edema (HAPE)

HAPE is an acute pulmonary disorder triggered by a hypoxic high-altitude environment, and it predominantly develops in high-altitude regions characterized by marked reductions in atmospheric pressure and oxygen partial pressure [116]. Its principal pathogenic factor stems from prolonged exposure to hypoxic high-altitude conditions; in the absence of timely intervention, this condition may progress to chronic hypoxic pulmonary hypertension. Traditional herbal preparations, including ginseng, act on multiple pharmacological targets and have demonstrated considerable application potential for the prevention and clinical management of HAPE [117,118]. Under hypobaric hypoxic conditions, the body is susceptible to oxidative stress and iron metabolic disorders, with subsequent induction of ferroptosis that represents a core mechanistic contributor to HAPE pathogenesis. Accumulating evidence from prior studies has verified that GRg3 attenuates hypoxia-induced tissue damage via diverse mechanisms [119]. He et al. [82] identified that GRg3 upregulates iron storage protein expression while downregulating transferrin receptor (TFRC) expression. This dual regulatory action preserves intracellular iron homeostasis, potently antagonizes ferroptosis induced by high-altitude hypoxia and attenuates acute pulmonary edema triggered by hypobaric hypoxia.

#### 2.3.6. Chronic Obstructive Pulmonary Disease (COPD)

COPD constitutes a prevalent, preventable respiratory disorder [120]. Chronic tobacco smoking acts as a primary contributor to COPD onset and progression. Smoking raises the risk of ERS in COPD patients [121] and triggers ferroptosis in bronchial epithelial cells [122]; ERS suppression accordingly mitigates cigarette smoke-driven ferroptosis within these cells [123]. Accumulating evidence verifies that ferroptosis represents a core pathogenic mechanism in COPD [124], a feature underpinned by the following pathological cascade: cigarette smoke initiates ferritinophagy, which facilitates free iron liberation and excessive lipid peroxidation alongside reduced expression of the antioxidant protein GPX4. In combination, these pathological changes propel ferroptosis and provoke ensuing inflammatory responses, directly contributing to the full pathological course of COPD. Ginsenoside Rg1 (GRg1) displays diverse biological activities, including anti-inflammatory [125] and anti-tumor effects [126]. Of note, GPX4 functions as a negative regulator of ferroptosis, and cigarette smoke downregulates GPX4 expression [127], which means reduced GPX4 expression directly initiates ferroptosis. Tan et al. [83] demonstrated that GRg1 efficiently restores antioxidant protein (e.g., GPX4) and GSH levels, with concomitant suppression of the protein kinase R-like endoplasmic reticulum kinase (PERK)/activating transcription factor 4 (ATF4) signaling pathway and the associated ERS. These regulatory influences markedly reduce ferroptosis, with attendant attenuation of COPD progression alongside amelioration of emphysematous lesions and pulmonary inflammatory responses.

#### 2.3.7. Ulcerative Colitis (UC)

UC is a chronic inflammatory disorder driven by a multitude of contributing factors, including genetic susceptibility, aberrant immune regulation, environmental exposure and intestinal mucosal barrier dysfunction. Lesions associated with UC predominantly affect the rectal and colonic mucosa [128], and affected patients typically manifest classic clinical symptoms including abdominal pain, diarrhoea and hematochezia. In current clinical practice, 5-aminosalicylic acid (5-ASA) preparations represent the primary therapeutic intervention for patients with mild-to-moderate active UC [129]. Yet clinical administration of these agents carries inherent risks, as they are capable of eliciting treatment-associated adverse events, including pancreatitis and cardiotoxicity [130], with consequent limitations imposed on their clinical applicability to a measurable extent. This clinical challenge highlights the necessity of developing novel therapeutic agents with enhanced targeting capacity, superior selectivity and reduced adverse effects as a core research priority for the future clinical management of UC. Emerging research evidence has verified the occurrence of ferroptosis within colonic tissues obtained from UC patients and corresponding experimental animal models [131]. Excessive iron overload is recognized as a core factor driving the pathological progression of UC [132]. Prior research has firmly established that GRh2 possesses diverse pharmacological activities, including anti-inflammatory and anti-tumor properties [133,134]. Zhao et al. [84] demonstrated that in a dextran sulfate sodium (DSS)-induced murine model of UC, GRh2 elevates miR-125a-5p expression and exerts specific inhibitory effects on the expression of its downstream target gene, a transcriptional activator. This regulatory mechanism mitigates ferroptosis activity within colonic tissues, with concomitant effective amelioration of UC-associated clinical symptoms.

#### 2.3.8. Neurodegenerative Disease (NDDs)

Chronic neuroinflammation has been identified as the core pathological pivot of neurodegenerative diseases, including Alzheimer’s disease (AD) and Parkinson’s disease (PD). It persists throughout the entire course of the disease and serves as a key driver of disease progression. [135]. In the pathological progression of neurodegenerative diseases, ROS produced by oxidative stress activate glial cells, which in turn secrete significant levels of pro-inflammatory cytokines (e.g., IL-1β and IL-6), thereby triggering neuroinflammation [136]. Crucially, once the aforementioned process is initiated, neuroinflammation conversely elevates oxidative stress levels, forming a positive feedback loop that amplifies the accumulation of oxidative stress. This ultimately leads to iron overload and ferroptosis, which is attributed to the dysregulated iron metabolism in disease-susceptible brain regions [137]. Accumulating evidence has verified a close association between ferroptosis and NDDs such as AD and PD [138]. GRg1 has exhibited promising intervention potential in this field [139]. Kong et al. [85] systematically verified that GRg1 can target neuroinflammation at its root by inhibiting the activation of the absent in melanoma 2 (AIM2) inflammasome via in vitro and in vivo experiments. Concurrently, GRg1 activates the signaling pathway of NRF2, a cytoprotective transcription factor. These two effects exert a synergistic action to downregulate the expression of ferroptosis suppressor proteins (e.g., GPX4 and FSP1), while concomitantly repressing the expression of iron uptake proteins [divalent metal transporter 1 (DMT1)/transferrin receptor (TfR)]. This markedly reduces lipid peroxidation levels, thereby effectively blocking neuronal ferroptosis, interrupting the vicious cycle between neuroinflammation and oxidative stress, and ultimately ameliorating inflammation-induced behavioral deficits and neuronal damage. The dual targeting of the AIM2 inflammasome and the NRF2 pathway by GRg1 highlights a key upstream intervention point: by resolving inflammation, GRg1 indirectly normalizes iron metabolism and redox balance, which are the true proximal drivers of ferroptosis.

#### 2.3.9. Sepsis

Sepsis is a life-threatening organ dysfunction triggered by the host’s dysregulated response to infection [140], involving the activation of pro-inflammatory and anti-inflammatory responses, as well as perturbations in non-immune pathways (e.g., metabolic, coagulation, neurological, hormonal, cardiovascular, and autonomic pathways). It frequently leads to dysfunction of multiple organs, including the heart and kidneys. Notably, in the cecal ligation and perforation (CLP)-induced sepsis model, the host exhibits typical ferroptosis features, accompanied by a significant upregulation of HO-1—a hub gene associated with ferroptosis regulation. Accumulating evidence has confirmed that GRb1 can markedly downregulate HO-1 expression and inhibit ferroptosis both in vitro and in vivo [141]. Specifically, GRb1 effectively reduced HO-1 protein levels; with the downregulation of HO-1, key ferroptosis-related indicators exhibited corresponding changes: decreased levels of iron ions and MDA, alongside restored GSH levels. Concurrently, the protein expression of GPX4 was upregulated. These findings collectively indicate that GRb1 blocks the ferroptosis process in sepsis by targeting HO-1.

Ferroptosis has been demonstrated to promote cardiomyocyte death and exacerbate cardiac dysfunction [142]. Currently, specific intervention strategies for sepsis-induced myocardial dysfunction (SI-MD) remain insufficient. GRg1 has been proven to possess extensive biological activities and prominent pharmacological effects [143,144], including cardio-protective and neuroprotective properties, which can effectively mitigate damage to cardiomyocytes and renal cells [145,146]. As a key signaling pathway, PI3K/AKT can reduce the transcriptional activation of pro-apoptotic genes, Bcl-2 interacting mediator of cell death (BIM), and p53 upregulated modulator of apoptosis (PUMA) [147] upon activation. This further inhibits the expression of FOXO3A—a downstream effector of AKT—and reduces cell apoptosis. Lin et al. [86] found that GRg1 could synergistically reduce cardiomyocyte apoptosis, inflammatory responses, and ferroptosis by activating the focal adhesion kinase (FAK)/AKT pathway and inhibiting FOXO3A expression, ultimately alleviating sepsis-induced myocardial injury.

Sepsis-induced acute kidney injury (SI-AKI) represents a common, life-threatening complication among critically ill patients [148] and carries a comparatively high mortality rate. At present, clinical therapeutic regimens for SI-AKI are lacking in specificity, with renal replacement therapy serving as the primary supportive intervention for affected individuals [149]. The pathogenic mechanisms underlying SI-AKI have not been fully elucidated, and involve a multitude of contributing factors, including uncontrolled inflammatory responses, metabolic dysregulation and impaired renal microcirculation [150]. GRg1 possesses diverse pharmacological properties, including anti-inflammatory, antioxidant and immunomodulatory activities [151], with notable therapeutic potential for the management of sepsis and its associated renal injury. Previous investigations have established that GRg1 attenuates inflammatory responses and apoptotic activity in lipopolysaccharide (LPS)-stimulated renal tubular epithelial cells, via activation of the PI3K/AKT pathway and concomitant suppression of the Nuclear Factor-κB (NF-κB) signaling pathway [152]. Further, GRg1 elicits renal protective effects through activation of the NRF2 pathway [153]. FSP1 acts as a key GPX4-independent anti-ferroptosis protein [154], which scavenges lipid peroxides via the Coenzyme Q_10_-Nicotinamide Adenine Dinucleotide (Phosphate) Hydrogen (CoQ_10_-NAD(P)H) pathway to mediate the suppression of cellular ferroptosis. Guo et al. [87] reported that GRg1 activates the FSP1-CoQ_10_-NAD(P)H pathway to enhance cellular resistance to lipid peroxidation, with attendant attenuation of ferroptosis in renal tubular epithelial cells, thereby alleviating the progression of SI-AKI.

## 3. An Integrative Framework: Core Intrinsic Mechanisms of Ginsenoside-Regulated Ferroptosis and Disease-Specific Downstream Pathways

The preceding review demonstrates that ginsenosides regulate ferroptosis through diverse signaling pathways across different disease models. However, a critical question remains: is there a common biological basis underlying these seemingly disparate mechanisms? Based on a systematic synthesis of the current literature, we propose an integrative framework positing that ginsenosides may primarily act upon the fundamental physicochemical state of the cell—including the modulation of iron metabolism, dysregulation of antioxidant systems, disruption of iron homeostasis, remodeling of lipid metabolism coupled with resultant peroxidation storms, and autophagic activity. These interconnected processes constitute the core intrinsic mechanisms of ferroptosis. Subsequently, distinct cell types, governed by their inherent metabolic profiles and stress thresholds, differentially activate or inhibit specific downstream signaling pathways. This context-dependent response ultimately leads to the opposing outcomes of ferroptosis promotion or inhibition under specific pathological conditions.

As presented in Table 4, we have categorized the effects of various ginsenosides according to these core intrinsic mechanisms of ferroptosis. This classification reveals several critical patterns:

First, the regulatory control of the iron metabolic system. For instance, the modulation of iron metabolism—whether through Rg5-mediated promotion of ferritinophagy to release labile iron, or through Rg3-mediated downregulation of transferrin receptor expression to restrict iron uptake—serves as a common ferroptosis trigger. However, the ultimate effect is profoundly cell-context dependent: in cancer cells, iron overload drives lipid peroxidation and cell death, whereas in hypoxically damaged tissues, restricting iron accumulation confers cytoprotection.

Second, the bidirectional modulation of antioxidant systems constitutes a central regulatory hub. Key antioxidant axes—including the NRF2/GPX4 axis, the SLC7A11/System Xc- system, and the FSP1/CoQ10 pathway—are recurrently targeted across diverse diseases, yet the direction of modulation is diametrically opposed. In tumor models, ginsenosides tend to suppress these antioxidant systems to induce ferroptosis in cancer cells. Conversely, in tissue injury models, they tend to activate these systems to alleviate oxidative stress-induced damage. This suggests that ginsenosides do not merely “activate” or “inhibit” a given pathway in a binary fashion, but rather perform a “calibration” function in accordance with the cellular stress state.

Third, the interplay between lipid metabolism remodeling and inflammation-cell death crosstalk constitutes a key synergistic mechanism. The synthesis of lipid peroxidation substrates mediated by ACSL4, the inflammation-mitochondrial injury axis mediated by the cGAS-STING pathway, and the crosstalk between the AIM2 inflammasome and NRF2 signaling all exemplify the capacity of ginsenosides to achieve nuanced regulatory control by integrating multi-layered signals.

## 4. Summary and Prospect

Ginsenosides, as natural active products, have had their anti-tumor and tissue protective mechanisms initially clarified. A critical observation from the reviewed literature is that ginsenosides exhibit a bidirectional regulatory effect on ferroptosis: they promote ferroptosis in cancer cells and activated hepatic stellate cells, yet inhibit it in the context of acute tissue injuries such as ischemia–reperfusion or sepsis. This dichotomy suggests that ginsenosides do not act as simple on/off switches for a single pathway, but rather function as context-dependent modulators of fundamental cellular states, including iron metabolism, redox balance, and metabolic activity. The disparate downstream signals—PI3K/AKT, NRF2, p53, or miRNAs—likely represent cell-type-specific and disease-specific responses to these upstream perturbations.

However, it is crucial to critically assess the strength of the evidence supporting these claims. A significant portion of the cited studies define ferroptosis primarily based on changes in biochemical markers such as MDA, ROS, GSH, and GPX4 expression. While informative, these markers are not exclusive to ferroptosis; they are also indicative of general oxidative stress and can accompany other forms of cell death. According to current ferroptosis research standards, rigorous validation requires functional rescue experiments using specific ferroptosis inhibitors (e.g., ferrostatin-1, liproxstatin-1) or iron chelators (e.g., deferoxamine). The field would greatly benefit from future studies that incorporate these genetic and pharmacological interventions to definitively confirm the causal role of ferroptosis in ginsenoside-mediated effects and to rule out contributions from other regulated necrosis pathways.

Moreover, the biological effects of the same ginsenoside component in regulating ferroptosis may be completely opposite in different disease contexts. For instance, in the liver fibrosis disease model, GRg3, GRb1 and GRh2 all exhibit the effect of promoting ferroptosis. However, in the myocardial ischemia/reperfusion and high-altitude pulmonary edema disease models, GRg3 exerts a protective effect on the body by inhibiting the occurrence of ferroptosis. Similarly, GRb1 also demonstrated inhibitory effects on ferroptosis in the hypoxic–ischemic brain injury model and GRh2 in the ulcerative colitis model. The core regulatory factors of this bidirectional regulation of ferroptosis by the same component in different disease backgrounds remain unclear and require further exploration. Understanding the baseline cellular state—specifically the labile iron pool, the lipid composition of membranes, and the metabolic preference for glucose or glutamine—may hold the key to predicting whether a ginsenoside will act as an inducer or inhibitor of ferroptosis.

This review summarizes that the various active components extracted from ginseng, such as ginsenosides GRg1, GRg3, GRg5, GRh2, and GRh4, can target and intervene in core signaling targets or key regulatory pathways of ferroptosis, like NRF2, GPX4, SLC7A11, and FSP1. Ginsenosides play a precise regulatory role in the pathological processes of various diseases such as multiple cancers, liver fibrosis, neurodegenerative diseases, and organ ischemia–reperfusion injury. Moreover, this regulation is no single. We have found that ginsenosides can exhibit completely inconsistent regulation in different disease models. This different regulatory effect is specifically manifested in cancer models Ginsenosides can induce ferroptosis in tumor cells to achieve targeted killing, thereby achieving the effect of alleviating cancer progression. In normal tissue damage models, ginsenosides exert a tissue-protective effect by inhibiting ferroptosis, ultimately alleviating the damage caused by these diseases. It is worth noting that in diseases with the potential for precancerous lesions, such as liver fibrosis, ginsenosides inhibit the activation of HSCs by inducing ferroptosis, thereby improving the progression of liver fibrosis. This demonstrated that ginsenosides harbor bidirectional regulatory activities in targeting ferroptosis across distinct disease models. It further underscores the distinctive merits and translational potential of ginsenosides in mediating divergent therapeutic outcomes tailored to specific disease settings.

Of note, we observed that ginsenosides mediate regulatory actions in diverse disease models via core mechanisms centered on multi-pathway crosstalk—including ferroptosis-autophagy, ferroptosis-apoptosis, ferroptosis-pyroptosis, and ferroptosis-immunity crosstalk. Additionally, the potential function of ginsenosides in emerging research areas linked to ferroptosis, such as autoimmune diseases and metabolic disorders, deserves deeper exploration.

Despite the promising preclinical findings, several critical translational hurdles must be addressed before ginsenosides can advance to clinical applications targeting ferroptosis. First, the dose–response relationship of ginsenosides in ferroptosis modulation remains poorly characterized. The bidirectional effects observed—ferroptosis promotion versus inhibition—may be highly dose-dependent, with narrow therapeutic windows that vary across disease contexts. Establishing clear pharmacodynamic parameters and defining the threshold concentrations required for ferroptosis modulation in specific tissues is essential for future clinical trial design.

Second, the pharmacokinetic limitations of ginsenosides pose significant challenges. Natural ginsenosides generally exhibit poor water solubility, low intestinal absorption efficiency, rapid metabolism, and extensive first-pass effect, resulting in poor oral bioavailability. The concentrations achieving ferroptosis modulation in vitro often far exceed those attainable in vivo following conventional administration. Future research should prioritize the development of novel nano-delivery systems (e.g., liposomes, polymeric nanoparticles, lipid-based formulations) and structural modifications (e.g., glycosylation optimization, prodrug design) to enhance stability, improve bioavailability, and achieve targeted delivery to specific tissues or cell types.

Third, a substantial translational gap exists between preclinical ferroptosis markers and clinically meaningful outcomes. Most current studies rely on biochemical endpoints (MDA, GSH, and iron content) in cell cultures or animal tissues. However, the correlation between these surrogate markers and patient-relevant outcomes—such as tumor regression, improved organ function, prolonged survival, or enhanced quality of life—remains largely unexplored. Future translational studies should incorporate clinically relevant endpoints and establish whether modulation of ferroptosis biomarkers translates into tangible therapeutic benefits.

Furthermore, the safety profile of chronic ferroptosis modulation requires rigorous evaluation. Given that ferroptosis plays physiological roles in immune function and tissue homeostasis, prolonged systemic inhibition or promotion of ferroptosis may carry unforeseen on-target toxicities. Organ-specific effects, potential impacts on immune surveillance, and long-term consequences of perturbing iron metabolism must be systematically assessed in appropriate preclinical models before advancing to clinical trials.

In the future, through systematic and in-depth research, the molecular mechanism of ginsenosides’ bidirectional regulatory effect on ferroptosis can be further understood. Integrating the proposed framework of core intrinsic mechanisms with advanced pharmacokinetic-pharmacodynamic (PK-PD) modeling will be essential to predict disease-specific responses and guide rational formulation design. It is imperative that future investigations move beyond correlative markers and adopt a more rigorous methodological framework, incorporating specific inhibitor studies and genetic models to delineate the precise molecular targets and to provide theoretical support for the precise targeted treatment of various components of ginsenosides in cancer and non-cancer diseases. Ultimately, bridging the gap between bench and bedside will require a concerted effort combining medicinal chemistry optimization, advanced drug delivery strategies, and well-designed translational studies that link ferroptosis modulation to meaningful clinical endpoints. This will promote ginsenosides to become effective adjuvant therapeutic drugs for various diseases as soon as possible, truly achieving the transformation from laboratory research to clinical application.

## 5. Materials and Methods

A preliminary literature search on ferroptosis and ginsenoside compounds was completed through the PubMed database. The search terms include “ginsenosides” and “ferroptosis” or “ginsenosides” and “drug delivery system”. Relevant literature published from 2020 to 2025 was retrieved in the PubMed database using the above-mentioned search terms. The search results were screened according to the inclusion criteria, which were English articles describing ferroptosis and ginsenoside compounds. In addition, another search was conducted using the terms “ferroptosis” or “ginsenosides” or “glioblastoma” or “hepatocellular carcinoma” or “gallbladder cancer” or “renal cell carcinoma” or “colorectal cancer” or “multiple myeloma” or “liver fibrosis” or “acute liver injury” or “hypoxic–ischemic brain damage” or “subarachnoid hemorrhage” or “myocardial ischemia–reperfusion” or “high-altitude pulmonary edema” or “chronic obstructive pulmonary disease” or “ulcerative colitis” or “neurodegenerative disease” or “Sepsis”, Literature published from 2012 to 2025 was retrieved in this search. The results were screened based on the inclusion criteria: English articles describing relevant research content. Studies meeting the above criteria were included after screening the titles, abstracts and full texts. After screening, 154 articles were finally included to further support our findings.

## Figures and Tables

**Table 1:** Overview of therapeutic targets and model of different ginsenoside components targeting ferroptosis in cancer models. NR3C1: nuclear receptor subfamily 3, group C, member 1. HSPB1: heat shock protein family B (small) member 1. NCOA4: nuclear receptor coactivator 4. FOXO: Forkhead Box O. FSP1: ferroptosis suppressor protein 1. circFOXP1: circular RNA FOXP1. miR-447a: microRNA-447a. NRF2: nuclear factor erythroid 2-related factor 2. Stat3: Signal Transducer and Activator of Transcription 3. p53: Tumor Protein p53. SIRT2: sirtuin 2. HCC: hepatocellular carcinoma. GBC: gallbladder cancer. RCC: renal cell carcinoma. CRC: Colorectal cancer. MM: multiple myeloma.

Ginsenoside Components	Structure	Target	Models	Dose and Time	Cancer
Rg5	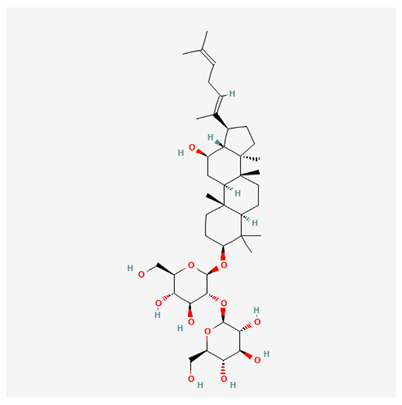	NR3C1/HSPB1/NCOA4 axis	Glioma stem cells	200 nM; 24 h	Glioblastoma [33]
CK	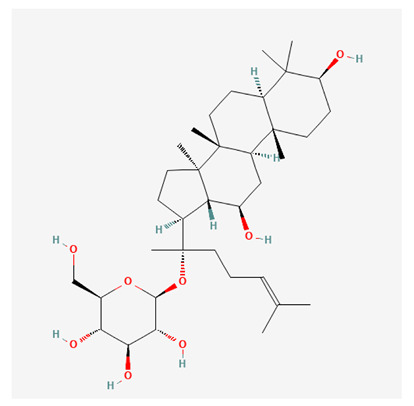	FOXO pathway	SK-Hep-1 cellHepG2 cell	40 μM; 48 h	HCC [34,35]
			HepG2-xenografted tumor-bearing nude mice	5/10/20 mg/kg/d; until the tumor volume reaches 1500 mm^3^	
RK1	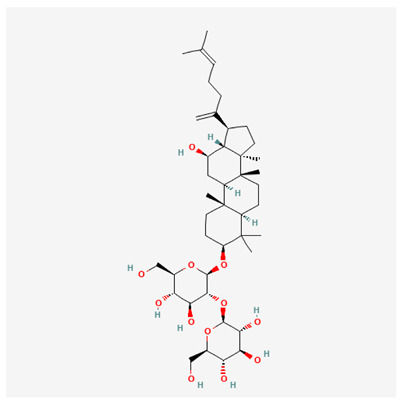	FSP1	HepG2 cell andHep3B cell	20 μM; 24 h	
Rg3	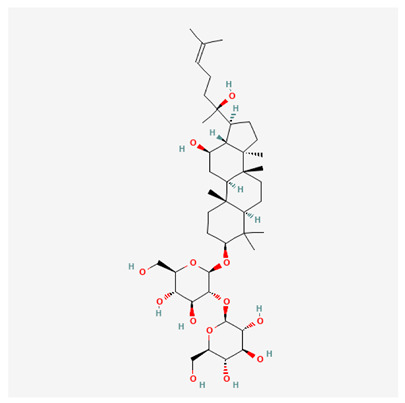	circFOXP1/miR-447a/PD-L1 axis	CBC cell	100 μM; 72 h	GBC [36]
Rh4	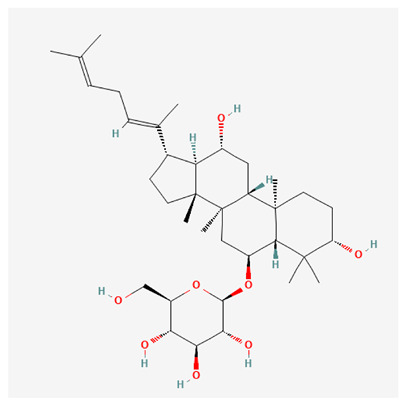	NRF2 pathway	786-O cell and ACH cell	100 μM; 24 h	RCC [37]
Rh3	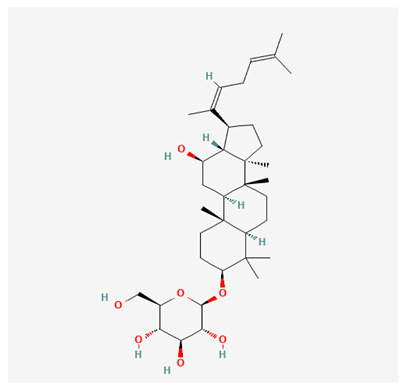	Stat3/p53/NRF2 axis	HCT116 cell andHT29 cell	40 μM; 48 h	CRC [38]
			BALB/c nude mice	20 mg/kg/d; 21 d	
Rh4	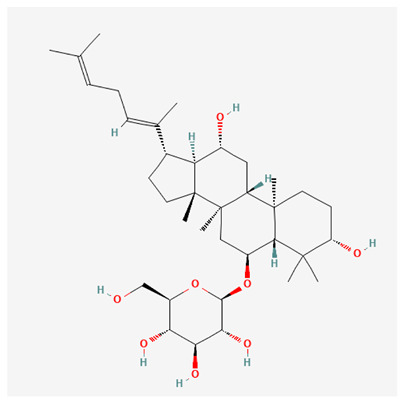	SIRT2 pathway	NCI-H929 cell	100 μM; 24 h	MM [39]

**Table 2:** Overview of therapeutic targets and models of different ginsenoside components targeting ferroptosis in hepatic fibrosis models. HSCs: hepatic stellate cells. BECN1: Beclin-1. SLC7A11: solute family 7 member 11. ACSL4: long-chain acyl-CoA synthase 4. IRF1: interferon regulatory factor 1. HK2: hexokinase 2. LPCAT3: Lysophosphatidylcholine Acyltransferase 3. ALOX5: Arachidonate Lipoxygenase 5.

Ginsenoside Components	Structure	Target	Model	Dose and Time	Disease
Rg3	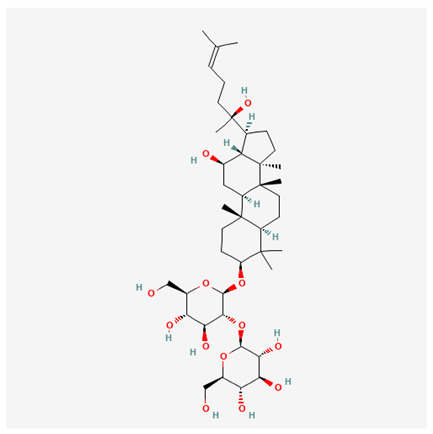	ACSL4 [71]	HSCs	20/40 μM; 24 h	liver fibrosis
			C57BL/6 male mice	10/20 mg/kg/d; oral gavage; once daily for 8 consecutive weeks	
Rb1	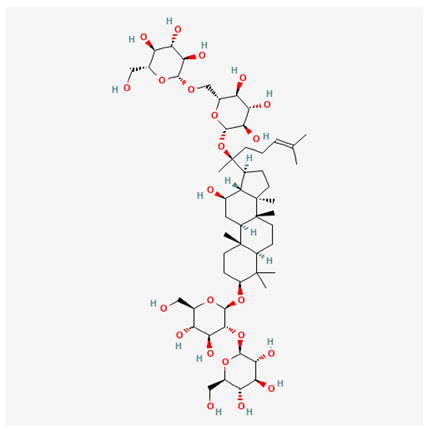	BECN1/SLC7A11 axis [73]	HSC cell and LX-2 cell	10/20 μM; 24 h	
			C57BL/6J male mice	10/20 mg/kg/d; oral gavage; once daily for 8 consecutive weeks	
Rh2	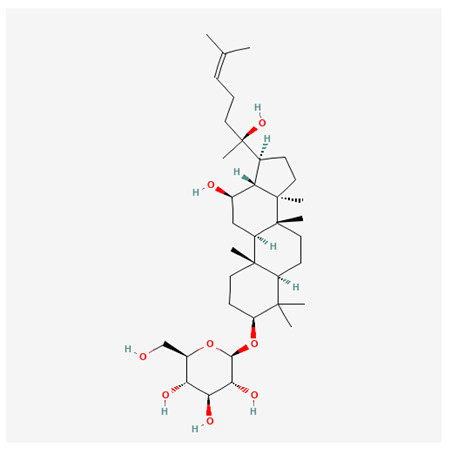	IRF1/SLC7A11 axis [74]	Primary HSC cell	10/20 μM; 24 h	
			C57BL/6J male mice	5/10 mg/kg/d; oral gavage; once daily for 8 consecutive weeks	
Rk1	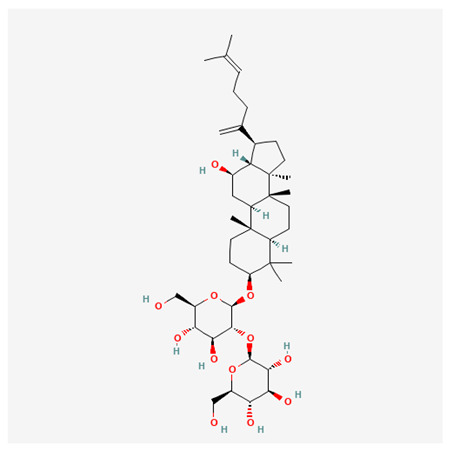	HK2/ACSL4/LPCAT3/ALOX5 pathway [75]	HSC-T6 cell and LX-2 cell	50 μM; 24 h	
			C57BL/6J male mice	10/20 mg/kg/d; oral gavage; once daily for 6 consecutive weeks	

**Table 3:** Overview of therapeutic targets and models of different ginsenoside components via the ferroptosis pathway in other non-cancerous disease models. NRF2: nuclear factor erythroid 2-related factor 2. LPS: lipopolysaccharide. cGAS: cyclic GMP-AMP Synthase. STING: Stimulator of Interferon Genes. CCl4: carbon tetrachloride. GPX4: glutathione peroxidase 4. DHODH: dihydroorotate dehydrogenase. keap1: Kelch-like ECH-associated protein 1. miR-144-3p: microRNA-144-3p. SLC7A11: solute family 7 member 11. PI3K: phosphatidylinositol 3-kinase. AKT: protein kinase B. PERK: protein kinase R-like endoplasmic reticulum kinase. ATF4: activating transcription factor 4. miR-125a-5p: microRNA-125a-5p. AIM2: melanoma 2. FAK: focal adhesion ki-nase. FOXO3A: Forkhead Box O3A. FSP1: ferroptosis suppressor protein 1. CoQ_10_-NAD(P)H: coenzyme Q_10_-Nicotinamide Adenine Dinucleotide (Phosphate) Hydrogen. ALI: Acute liver injury. HIBD: hypoxic–ischemic brain damage. SAH: subarachnoid hemorrhage. MI/R: myocardial ischemia–reperfusion. HAPE: high-altitude pulmonary edema. COPD: Chronic obstructive pulmonary disease. UC: Ulcerative colitis. NDDs: neurodegenerative disease. SI-MD: Sepsis-induced Myocardial dysfunction. SI-AKI: Sepsis-induced acute Kidney injury.

Ginsenoside Components	Structure	Target	Models	Dose and Time	Diseases
Rg5	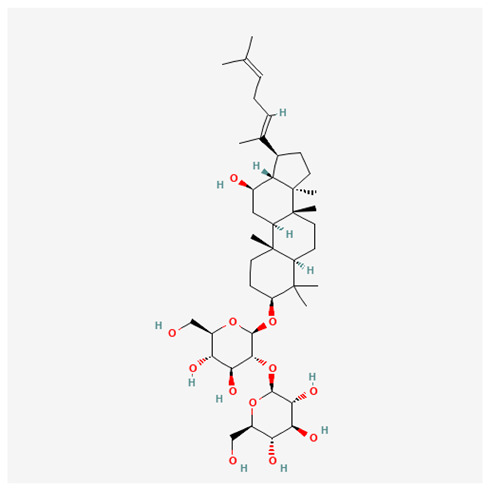	autophagy/NRF2/ferroptosis axis [76]	HpG2 cell	0.2/1/5 μM; 24 h	ALI
			C57BL/6 male mice (induced by LPS)	15/30 mg/kg (intraperitoneal injection; once daily for 3 consecutive days	
Rd	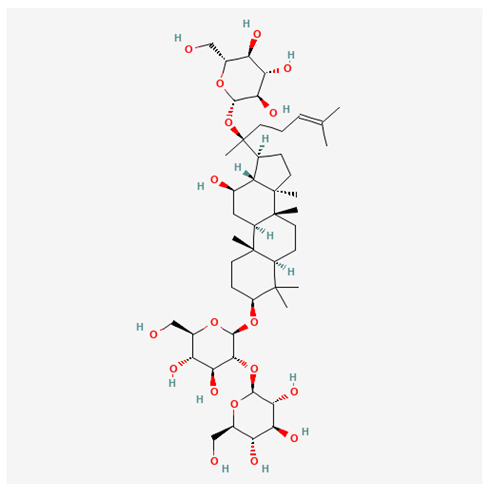	cGAS/STING pathway [77]	C57BL/6 male mice (induced by CCl4)	10/20 mg/kg; intraperitoneally injection; Twice (1 h before CCl_4_ and 1 h before sacrifice)	ALI
Rb1	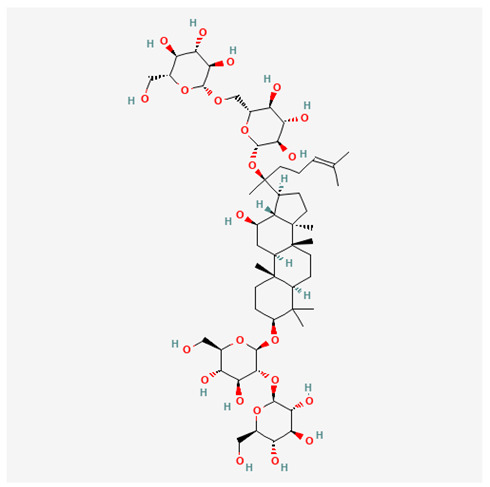	System Xc-GSH-GPX4 axis [78]	PC12 cell	1 mg/L; simultaneously with modeling treatment (after 12 h of hypoxia, followed by 2 h of subsequent culture)	HIBD
			neonatal SD rats	20 mg/kg/d; intraperitoneally injection; for 3 consecutive days	
Rd	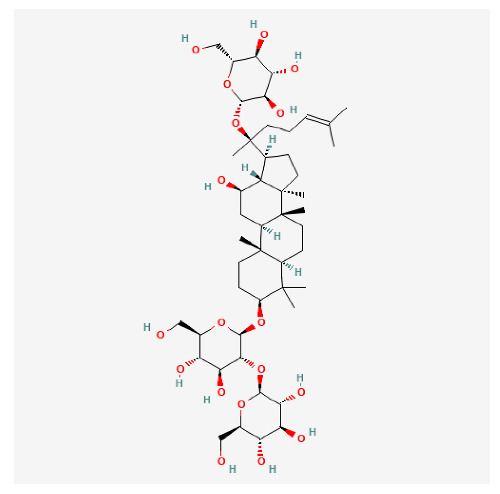	cGAS/STING/DHODH pathway [79]	HT22 cell	10 μM; once; simultaneously with modeling treatment	SAH
			SD male rats	60 mg/kg; intraperitoneal injection; twice (30 min before SAH and 1 h after SAH)	
Rg3	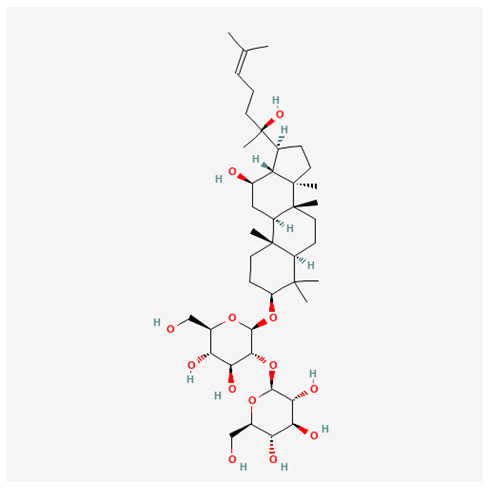	keap1/NRF2/GPX4 pathway	H9C2 cell	5/10/20 μM; 24 h	MI/R [80,81]
			C57BL/6 male mice	5/10/20 mg/kg; oral gavage; once daily for 7 consecutive days	
Re	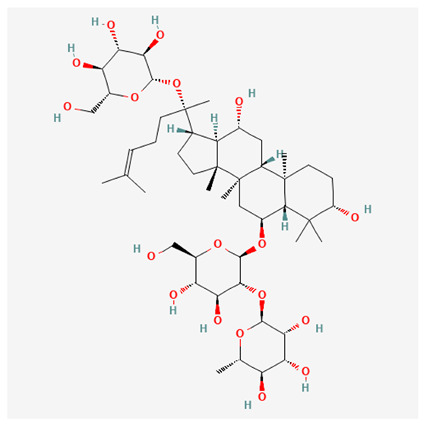	miR-144-3p/SLC7A11 pathway	WKY female rats	150 mg/kg/d; oral gavage; once daily for 5 consecutive days	
Rg3	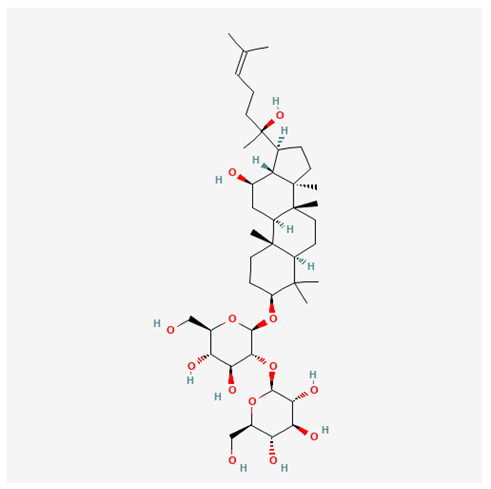	PI3K/AKT pathway	C57BL/6 male mice	15/30 mg/kg/d; intraperitoneally injection; once daily for 3 consecutive days	HAPE [82]
Rg1	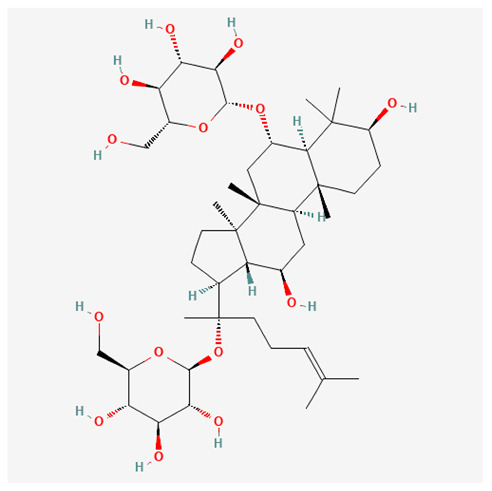	PERK/ATF4 axis [83]	BEAS-2B cell	40 μM; 24 h	COPD
			C57BL/6 male mice	10/20 mg/kg/d; oral gavage; once daily for 4 consecutive weeks	
Rh2	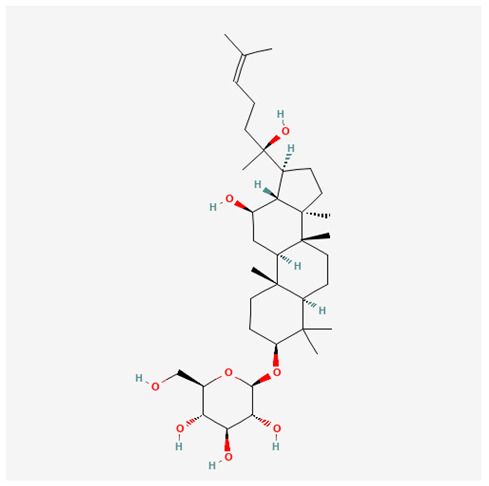	miR-125a-5p [84]	NCM460 cell	5/10 μM; pretreatment for 30 min, followed by subsequent treatment for 24 h	UC
			C57BL/6J male mice	50 mg/kg; oral gavage; once daily for 7 consecutive days	
Rg1	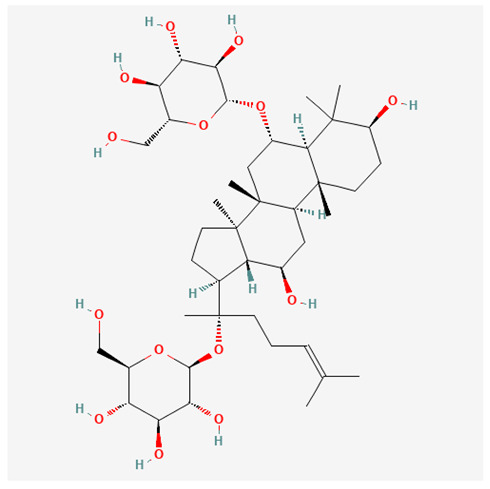	AIM2-NRF2 pathway [85]	HT22 cell	10 μM; 24 h	NDDs
			C57BL/6J male mice	5/10/20 mg/kg/d; intraperitoneally injection; once daily for 3 consecutive weeks	
Rg1	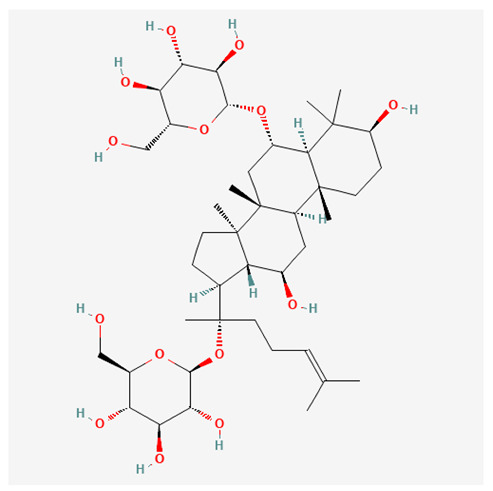	FAK/AKT-FOXO3A pathway [86]	H9C2 cell	25 μM; 6 h (post-LPS treatment)	SI-MD
			C57BL/6J male mice	35/70 mg/kg/d; intraperitoneally injection; once daily for 6 consecutive weeks	
		FSP1-CoQ_10_-NAD(P)H pathway [87]	HK-2 cell	150 μM; 24 h	SI-AKI
			SD male rats	50 mg/kg; intraperitoneal injection; twice (0.5 h and 12 h postoperatively)	

**Table 4:** An Integrative Framework of Ginsenoside-Mediated Ferroptosis Regulation: From Core Intrinsic Mechanisms to Disease-Specific Downstream Pathways.

The Core Mechanism of Ferroptosis	Ginsenoside/Specific Regulatory Mechanisms	Downstream Pathway/Effect	Disease Model	Outcome
Regulation of Iron Metabolism	Rg5: Binds to NR3C1, upregulates NCOA4, promotes ferritinophagy, and releases labile iron	Activation of NR3C1/HSPB1/NCOA4 axis	Glioblastoma	Promotion
	Rg3: Activates PI3K/AKT, upregulates iron storage proteins, downregulates transferrin receptor (TFRC), reduces iron uptake	PI3K/AKT pathway	HAPE	Inhibition
	Rb1: Targets HO-1, inhibits heme catabolism, reduces iron release	Inhibition of HO-1	Sepsis	Inhibition
Regulation of Redox Homeostasis	Rg5: Modulates autophagic flux (p62/LC3), restores NRF2 activity, scavenges ROS	Activation of p62/NRF2 axis (upregulation of HO-1, GPX4, FTH1)	ALI	Inhibition
	Rg3: Activates Keap1/NRF2/GPX4 pathway, scavenges mitochondrial ROS	Keap1/NRF2/GPX4 axis	MI/R	Inhibition
	Rh4: Inhibits NRF2 transcriptional activity, abrogates antioxidant defense	Inhibition of NRF2 (downregulation of GPX4)	RCC	Promotion
	CK: Inhibits PI3K/AKT, activates FOXO, downregulates SLC7A11/GPX4	PI3K/AKT inhibition and FOXO activation	HCC	Promotion
	Rb1: Activates System Xc^−^-GSH-GPX4 axis, enhances cystine uptake and glutathione synthesis	System Xc^−^-GSH-GPX4 axis	HIBD	Inhibition
	Rh2: Activates IRF1, transcriptionally inhibits SLC7A11, impairs cystine uptake	IRF1/SLC7A11 axis	Liver fibrosis	Promotion
	Re: Inhibits miR-144-3p, derepresses SLC7A11, restores cystine uptake	miR-144-3p/SLC7A11 axis	MI/R	Inhibition
	Rg1: Inhibits PERK/ATF4 pathway, alleviates ER stress, restores GPX4 expression	PERK/ATF4 axis	COPD	Inhibition
	Rg1: Activates FSP1-CoQ10-NAD(P)H pathway, enhances GPX4-independent antioxidant defense	FSP1-CoQ10-NAD(P)H axis	SI-AKI	Inhibition
Lipid Metabolism Remodeling	Rg3: Epigenetic regulation (inhibition of DNMT3B), demethylates ACSL4 promoter, increases substrates for lipid peroxidation	miR-6945-3p/DNMT3B/ACSL4 axis	Liver fibrosis	Promotion
	RK1: Binds to and stabilizes HK2, activates ACSL4/LPCAT3/ALOX5 axis, promotes lipid peroxidation	HK2/ACSL4/LPCAT3/ALOX5 axis	Liver fibrosis	Promotion
	Rh4: Inhibits SIRT2, upregulates ACSL4 while downregulating SLC7A11/GPX4/FTH1 (multi-target)	SIRT2 inhibition (ACSL4↑, SLC7A11/GPX4/FTH1↓)	MM	Promotion
Autophagy and Lysosomal Pathways	Rb1: Activates BECN1, promotes BECN1-SLC7A11 complex formation, enhances autophagy-dependent ferroptosis	BECN1/SLC7A11 axis	Liver fibrosis	Promotion
Crosstalk Between Inflammation and Cell Death	Rg3: Inhibits circFOXP1/miR-447a/PD-L1 axis, restores CD8^+^ T cell function, induces immune-mediated ferroptosis	circFOXP1/miR-447a/PD-L1 axis	GBC	Promotion
	Rd: Inhibits cGAS/STING pathway, attenuates inflammation-driven mitochondrial damage	cGAS/STING pathway	ALI	Inhibition
	Rd: Inhibits cGAS/STING/DHODH pathway, preserves mitochondrial function	cGAS/STING/DHODH axis	SAH	Inhibition
	Rg1: Inhibits AIM2 inflammasome while activating NRF2, downregulates iron uptake proteins (DMT1/TfR), upregulates GPX4/FSP1	AIM2 inhibition + NRF2 activation	NDDs	Inhibition
	Rg1: Activates FAK/AKT, inhibits FOXO3A, reduces apoptosis and inflammation	FAK/AKT-FOXO3A axis	SI-MD	Inhibition
Other/Multi-Target Integration	Rh3: Modulates Stat3/p53/NRF2 axis, concurrently induces ferroptosis and pyroptosis	Stat3/p53/NRF2 axis	CRC	Promotion
	RK1: Directly inhibits FSP1, blocks CoQ10 regeneration, disrupts membrane antioxidant capacity	FSP1 inhibition	HCC	Promotion
	Rh2: Activates miR-125a-5p, targets and inhibits transcription factor SP1, attenuates colonic ferroptosis	miR-125a-5p/SP1 axis	UC	Inhibition

## Data Availability

No new data were created or analyzed in this study. Data sharing is not applicable to this article.

## Abbreviations

The following abbreviations are used in this manuscript:

TCM	Traditional Chinese medicine
NSCLC	Non-small cell lung cancer
PD-L1	Programmed Death-Ligand 1
circ-0003074	circular RNA 0003074
miR-516b-5p	MicroRNA-516b-5p
KPNA4	Karyopherin Alpha 4
PCD	Programmed cell death
GSH	Glutathione
MDA	Malondialdehyde
ROS	Reactive oxygen species
GPX4	Glutathione peroxidase 4
HSCs	Hepatic stellate cells
GRg5	Ginsenoside Rg5
GRb1	Ginsenoside Rb1
GRg3	Ginsenoside Rg3
NR3C1	Nuclear receptor subfamily 3, group C, member 1
HSPB1	Heat shock protein family B (small) member 1
NCOA4	Nuclear receptor coactivator 4
HCC	Hepatocellular carcinoma
GRK1	Ginsenoside RK1
FSP1	Ferroptosis suppressor protein 1
GCK	Ginsenoside compound K
PI3K	Phosphatidylinositol 3-kinase
AKT	Protein kinase B
FOXO1	Forkhead Box O1
SLC7A11	Solute family 7-member 11
GBC	Gallbladder cancer
CD8^+^T	CD8-positive T
ERS	Endoplasmic reticulum stress
circFOXP1	Circular RNA FOXP1
miR-447a	MicroRNA-447a
RCC	Renal cell carcinoma
GRh4	Ginsenoside Rh4
NRF2	Nuclear factor erythroid 2-related factor 2
CRC	Colorectal cancer
GRh3	Ginsenoside Rh3
GSDMD	Gasdermin D
Stat3	Signal Transducer and Activator of Transcription 3
p53	Tumor Protein p53
MM	Multiple myeloma
SIRT2	Sirtuin 2
FTH1	Ferritin heavy chain 1
ACSL4	Long-chain acyl-CoA synthase 4
α-SMA	α-smooth muscle actin
ECM	Extracellular matrix
BECN1	Beclin-1
DNMT3B	DNA methyltransferase 3B
GRh2	Ginsenoside Rh2
IRF1	Interferon regulatory factor 1
HK2	Hexokinase 2
LPCAT3	Lysophosphatidylcholine Acyltransferase 3
ALOX	Arachidonate Lipoxygenase
ALI	Acute liver injury
AREs	Antioxidant response elements
LPS	Lipopolysaccharide
HO-1	Heme oxygenase-1
SQSTM1/p62	Sequestosome 1
LC3A/B	Microtubule-associated protein 1 light chain 3A/B
GRd	Ginsenoside Rd
CCl4	Carbon tetrachloride
cGAS	Cyclic GMP-AMP Synthase
STING	Stimulator of Interferon Genes
HIBD	Hypoxic–ischemic brain injury
SAH	Subarachnoid hemorrhage
TfR	Transferrin receptor
DHODH	Dihydroorotate dehydrogenase
EBI	Early brain injury
MI	Myocardial ischemia
PCI	Percutaneous coronary intervention
TAC	Transverse aortic coarctation
MI/R	Myocardial ischemia–reperfusion
Keap1	Kelch-like ECH-associated protein 1
GRe	Ginsenoside Re
HAPE	High-altitude pulmonary edema
TFRC	Transferrin receptor
COPD	Chronic obstructive pulmonary disease
GRg1	Ginsenoside Rg1
PERK	Protein kinase R-like endoplasmic reticulum kinase
ATF4	Activating transcription factor 4
UC	Ulcerative colitis
5-ASA	5-aminosalicylic acid
DSS	Dextran sulfate sodium
NDDs	Neurodegenerative disease
AD	Alzheimer’s disease
PD	Parkinson’s disease
AIM2	melanoma 2
DMT1	Divalent metal transporter 1
CLP	Cecal ligation and perforation
SI-MD	Sepsis-induced myocardial dysfunction
BIM	Bcl-2 interacting mediator of cell death
PUMA	p53 upregulated modulator of apoptosis
FAK	Focal adhesion kinase
SI-AKI	Sepsis-induced acute kidney injury
CoQ_10_-NAD(P)H	Coenzyme Q_10_-Nicotinamide Adenine Dinucleotide (Phosphate) Hydrogen
